# Medical genetics and genomic medicine in Turkey: a bright future at a new era in life sciences

**DOI:** 10.1002/mgg3.326

**Published:** 2017-09-03

**Authors:** Tayfun Özçelik

**Affiliations:** ^1^ Department of Molecular Biology and Genetics Bilkent University Ankara Turkey

## Abstract

Medical genetics and genomic medicine in Turkey: a bright future at a new era in life sciences.

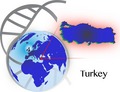

## Turkey – The Bridge

Turkey is a transcontinental country often described as the bridge between the Eastern and Western civilizations. Continental name etymologies for Europe and Asia refer to the lands in present day Turkey. Asia, attributed to Herodotus (about 440 BCE), refers first to the east bank of the Aegean Sea, and later to the present day “Anatolian peninsula” or “Asia Minor.” Europa, the name of the beautiful daughter of the Phoenician King Phoenix (about 2000 BCE), refers to the west banks of the Bosphorus, Dardanelles, and the Aegean Sea.

**Figure 1:**
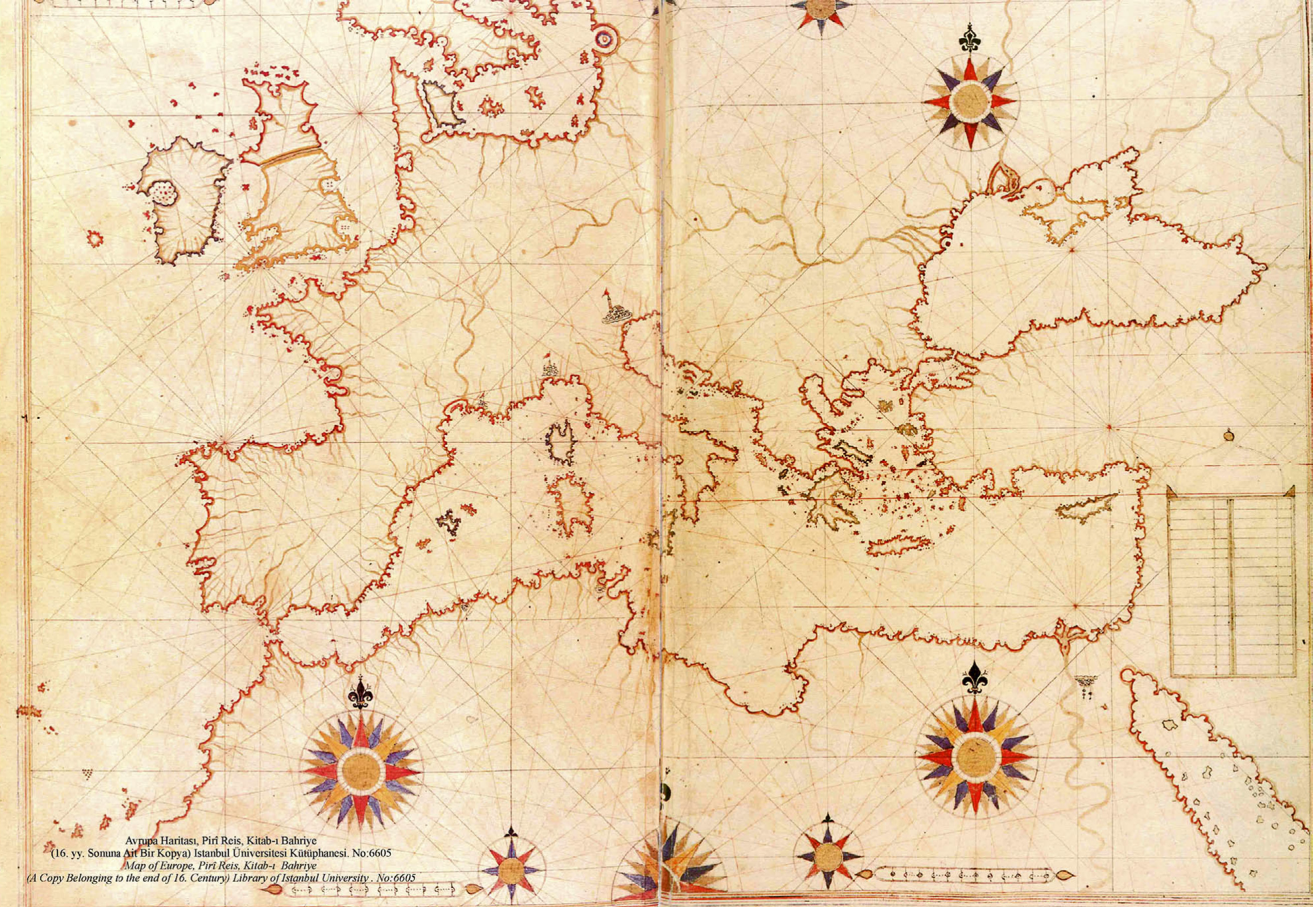
Map of the Mediterranean basin by Piri Reis, an Ottoman admiral, geographer, and cartographer. The map shown is from the 16th century book *Kitab‐ı Bahriye*.

Anatolian languages are the oldest branches of Indo‐European languages, spoken since at least the 19th century BCE. Beginning from the Bronze and Iron ages and encompassing the classical antiquity, the Ottoman Empire and present day Turkey, Anatolia has been home to many civilizations such as Hattians & Hurrians, Assyrians, Hittites, Greeks, Thracians, Phyrigians, Urartians, Armenians, and Turks. The legacy of these civilizations is very much present today, not only in archeological sites, traditions, literature, arts, and medicine, but also encoded in the genomes of the people that inhabit this genetically and geographically diverse land (Fig. 1).

The country has a very rich history. Some fascinating examples are:

Göbekli Tepe, the oldest known manmade religious structure dating to circa 10,000 BCE, lies between the Tigris and Euphrates rivers nearby the town of Urfa in the upper Mesopotamia.
According to Jewish and Muslim traditions, Urfa is the hometown of Abraham.
Earliest examples of paintings in history exist on a wall of a Çatalhöyük House dating from 6200 BCE.
Mount Ararat is in Anatolia. According to tradition, Noah's Arc landed on Mount Ararat.
Many city names originate in Anatolia, such as Philadelphia, Paris, Antioch, and Troy.
Two of the Seven Wonders of the World, Temple of Artemis and Mausoleum at Halicarnassus, are in Anatolia.
Alexander the Great cut the Gordian knot near Ankara.
Treaty of Kadesh, the first recorded international treaty, was enacted between Hittite and Egyptian Empires, Hattusilis III, and Ramses II in 1284 BCE.
King Midas was an Anatolian.
Lydians in Sardis were the first in history to issue coins in 640 BCE.
Julius Caesar famously said “Veni, vidi, vici.” (I came, I saw, I conquered) in 47 BCE in Amasya.
The seven churches of the Apocalypse are all situated in Anatolia.
St. Nicholas (Santa Claus) was born and lived as bishop of Myra in Demre.
For the first time in history, the Seljuks created state insurance for the losses of tradesmen.
The first man ever to fly was Turkish: Using two wings, Hezarfen Ahmet Celebi flew from the Galata Tower over the Bosphorus to land in Uskudar in the 17th century.

## Origins in History

Turkic peoples have Central Asian origin. We see references to Turks in the writings of Herodotus, the Greek historian who lived in the fifth century BCE in Halicarnassus (present day Bodrum, Turkey), and Pomponius Mela, the earliest Roman geographer who lived in Tingentera (present day Algeciras, Spain) during the first century CE. They refer to the “Turcae” or “Tyrcae” in the forests north of the Sea of Azov in Crimea. In Chinese sources, reference to Turks appeared during the 600s CE as nomadic traders in the south of the Altai Mountains. The term “Türk” or “Türük” was first documented circa eighth century in Central Asia in the earliest surviving Old Turkic Orkhon inscriptions of Göktürks (*Celestial Turks*). Turkey is derived from Medieval Latin *Turchia*. Byzantine emperor and scholar Constantine VII also used the name *Tourkia*, but this was a referral to Magyars. Arabs referred to the Mamluk Sultanate as *al‐Dawla al‐Turkiyya* (*State of Turkey*). European contemporaries sometimes referred to the Ottoman Empire as *Turkey* or the *Turkish Empire*.

Seljuk Turks entered into Anatolia in 1071 following the Battle of Manzikert against the Byzantine Empire and ruled the land until the Mongols’ Conquest of Anatolia in the 11th century. Following a short‐lived period of Beyliks (Turkish lords of Karasi, Saruhan, Aydin, Menteşe, Teke, Hamid, and Germiyan), a group of Oghuz Turks calling themselves Ottomans took control of Anatolia, first crossing the Dardanelles and then conquering Constantinople in 1453 under Sultan Mehmed II. The Ottoman Empire reached at the height of its power during the 16th and 17th centuries, under Suleiman the Magnificent, as a multinational and multilingual entity controlling much of Southeast Europe, parts of Central Europe, Western Asia, the Caucasus, North Africa, and the Horn of Africa.

*Konstantiniyye* – also referred to as *Stamboul* or *Islambol* or *Der‐i Sa’ãdet* (Abode of Felicity) or *Bãb‐i Álî* (The Sublime Port) or *Pâyitaht* (The Seat of the Throne) – as its capital, the Ottoman Empire ruled in the Mediterranean basin for six centuries until its decline in the late 18th and early 19th centuries mainly due to the weakening of the military system and falling behind that of their European rivals, the Habsburg and Russian Empires. At the end of World War I, the Empire lost most of its Balkan and Middle Eastern territories leading to the creation of Balkan and modern Arab countries, and the Republic of Turkey.

Mustafa Kemal Atatürk and his colleagues founded modern Turkey in 1923 after the Turkish War of Independence (1919–1922). The country has borders with Greece, Bulgaria, Georgia, Armenia, Azerbaijan, Iran, Iraq, and Syria. Turkey is a charter member of the UN, an early member of NATO, and a founding member of the OECD, OSCE, OIC, and G‐20.

## Turkish Science in the Wake of World War II

In order to understand the journey of life sciences and genetics in Turkey, it is necessary to take a look at the key events that shaped the Turkish medical establishment and research community during the Ottoman modernization of *Tanzimat* in the 19th century and the early days of the Republic. *Darülfünun*, the predecessor of Istanbul University, is linked to Emperor Justinian's sixth century codification of Roman law, the *Corpus Iuris Civilis*, marking the commencement of teaching and paving the way to the establishment of medieval universities such as Bologna (1088), Paris (c.1150), and Oxford (1167). With the conquest of Constantinople, scholarship continued at the *Fatih Medrese*, and expanded to *Mekteb‐i Tıbbiye‐i Şahane* (Magnificent School of Medicine) in the 19th century. *Mühendishane‐i Bahr‐i Hümayun* (School of Naval Engineering) of the 18th century evolved to Istanbul Technical University. There is then the first Turkish Academy of Sciences, *Encümen‐i Daniş*, established in 1860s to live only for a decade and reemerge approximately one century later with the establishment of TÜBİTAK (The Scientific and Technological Research Council of Turkey) and TÜBA (Turkish Academy of Sciences).

**Figure 2:**
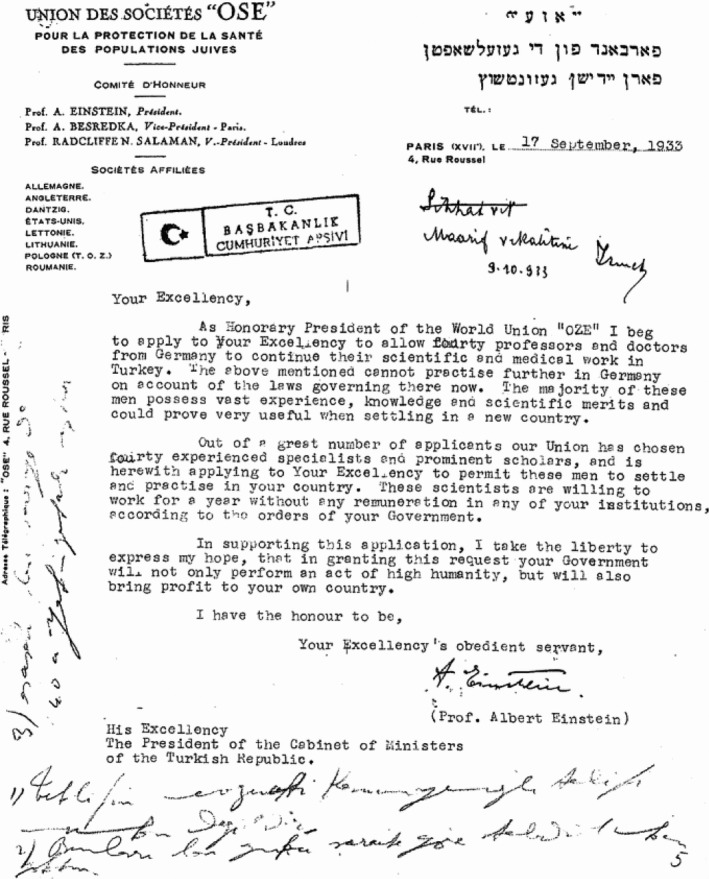
Albert Einstein's letter to the Turkish government in 1933.

In 1933, Einstein wrote a letter to Atatürk asking for his permission to accept scientists (mainly but not restricted to physicians) who were oppressed under the Third Reich (Fig. 2). Atatürk and İnönü, the Prime Minister and the second President of Turkey, welcomed this request and initiated the far‐reaching “University Reform of 1933,” which resulted in the re‐establishment of Istanbul University followed by Ankara, Istanbul Technical, Ege, Hacettepe, Middle East Technical, Bilkent, and now more than 100 institutions of higher education. The University Reform of 1933 marks the beginning of modern university establishments in Turkey.

More than 80 professors and doctors of medicine arrived during the next decade including Erich Frank (surgery), Phillip Schwartz (medicine), Ernst Eduard Hirsch (law), Fritz Neumark (economy), Hans Winterstein (physiology), Leo Brauner (botanics), Hans Willebrandt (agriculture), Rosa Maria Rössler (pathology), Fritz Arndt (chemistry), Albert Eckstein (pediatrics), Ernst Reuter (urban planning, later the German mayor of West Berlin during the cold war), Werner Laquer (obstetrics and gynecology), Friedrich Reimann (genetics), Clemens Holzmeister (architect, planned the Grand National Assembly of Turkey), Felix Michael Haurowitz (biochemistry), Hugo Braun (microbiology), Siegfried Oberndorfer (pathology), Rudolf Nissen (surgery), and others. At one point, nearly 35% (85/237) of the entire faculty of Istanbul University was composed of foreign scientists. We can now trace these names to the nominators from Turkey at the Nobel Nomination Database.

## Beginnings: Clinical Observations that Lead the Way to Basic Research

Human genetics first flourished through clinical description of genetic syndromes followed by gene identification studies mainly in consanguineous families. Examples include Say syndrome (181180) and Barber‐Say syndrome (#209885, BBRSAY) by Burhan Say; Cenani‐Lenz syndactyly syndrome (#212780, CLSS) by Asim Cenani; Tukel syndrome (609428; CFEOM4) by Turgut Tükel; Syndactyly, Malik‐Percin type (#609432, MSSD) by Ferda Perçin; Cerebellar ataxia, mental retardation, and disequilibrium syndrome (#224050, CAMRQ1; #613227 CAMRQ2; #610185) CAMRQ3; #615268 CAMRQ4) by Üner Tan and Tayfun Özçelik; Primary intraosseous vascular malformation (**#**606893, VMOS) by Nurten Akarsu. A comprehensive list has been presented elsewhere (Özçelik et al. 2010). Harmonious collaborations were instrumental in these studies (Editorial, 2009, 2010).

**Figure 3:**
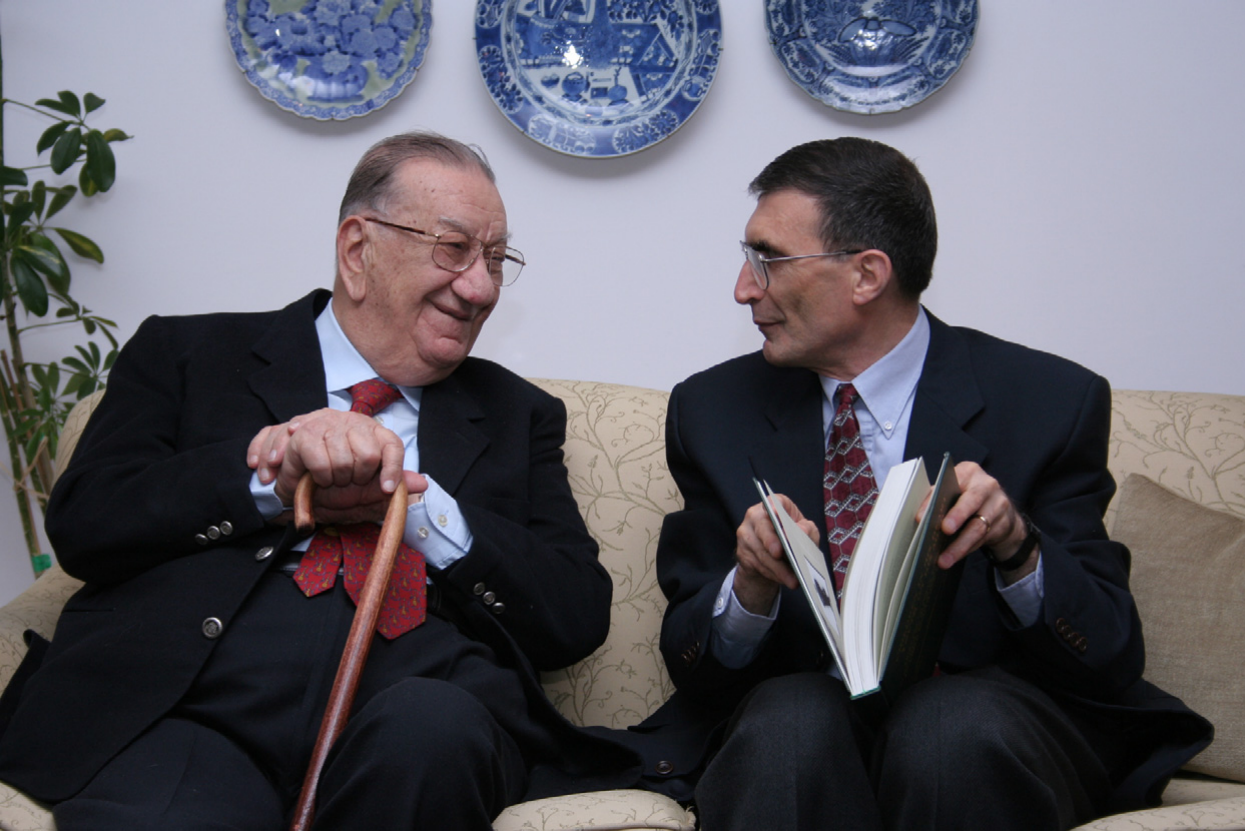
Aziz Sancar and İhsan Doğramacı discussing DNA repair.

But it is İhsan Doğramacı's remarkable tenure as an architect of higher education and child health, and Aziz Sancar's relentless commitment to scientific inquiry that have the farthest reaching effects on the human genetics community in Turkey (Fig. 3).

İhsan Doğramacı was born during World War I to an affluent Turkmen family in the Ottoman Empire province of Erbil (present day Iraq). He was one of the founders of WHO, the longest serving Executive Board member of UNICEF and a tireless campaigner for world peace. Through his long tenure first as a scientist–physician (Dogramaci 1946; Gocmen et al. 1986) then as a statesman (http://www.bilkent.edu.tr/hocabey/indexeng.html), he established several major universities and centers of excellence, and laid the foundations for the advancement of human genetics and genomics in Turkey.

Aziz Sancar was born in the southeastern town of Savur, Mardin shortly after the end of World War II. His chief mentor at Istanbul Medical School was Muzaffer Aksoy, who established the link between benzene exposure and risk of leukemia. He also defined several hemoglobin mutations including the one named “Istanbul.” His other mentors include Mutahhar Yenson (biochemist), Sevim Büyükdevrim (diabetologist), Nuran Gökhan (physiologist), Türkan Erbengi (histologist), Müfide Küley (one of the first Turkish female internists), Remzi Özcan (cardiologist) who were among the first generation of scientists trained by scholars who had fled Nazism (Aksoy 1955, 1962; Aksoy and Lehmann 1957; Devrim and Recant 1966; Erdem et al. 1966; Aksoy et al. 1972).

It was in the mid‐90s that Aziz Sancar reunited with the Turkish academia after going to the United States in the early 70s to pursue a career in science. He was already established as a leading figure in DNA repair. And a real breakthrough came in 1996 during a transatlantic flight when Dr. Sancar was returning from his annual pilgrimage to Turkey. Sleepless, he stumbled on an article in the airline magazine on transcontinental journeys, its health effects, and jetlag. He began reading through and learned for the first time the Latin origins of the term *circadian rhythm* (circa: about and dies: days). Humans have it, animals have it, and plants have it – a more or less 24‐hour inner clock that regulates all cellular functions. Could this be the answer to a more than a decade‐old mystery, the true biological function of one of the first ever identified components of DNA repair, *photolyase*? This enzyme, cloned by Dr. Sancar during his Ph.D. studies in the early 70s, repairs UV‐damaged DNA in bacteria. It was the first player identified in the quest to solve the mechanistic properties of nucleotide excision repair. Ironically, *photolyase* is found in many organisms including marsupials but not placental mammals; so, what was this photolyase homolog doing in humans and mice? Dr. Sancar, after reading the flight magazine article, suspected that it might function in the circadian clock that he later described as one of the most exciting eureka moments of his career (Zagorski 2005). *Photolyase* is one of the critical components in the regulation of circadian rhythm. The rest is history (Hsu et al. 1996; Miyamoto and Sancar 1998). The genetics community in Turkey eagerly waited to hear the name of Aziz Sancar every year that the Nobel prizes were announced. But the grand dame of the Turkish academic institutions, Istanbul University, was resourceful and the first ever Nobel laureate from Turkey proved to be another of her graduates, Orhan Pamuk. This was 2006 and it was in the field of literature. And the climax of the Turkish science was reached on October 8, 2015 when the Royal Swedish Academy of Sciences decided to award the Nobel Prize in Chemistry for 2015 to Tomas Lindahl, Paul Modrich, and Aziz Sancar “for mechanistic studies of DNA repair.”

## A Nobel Medal at Anıtkabir: the Nation's Most Important Memorial and Museum

**Figure 4:**
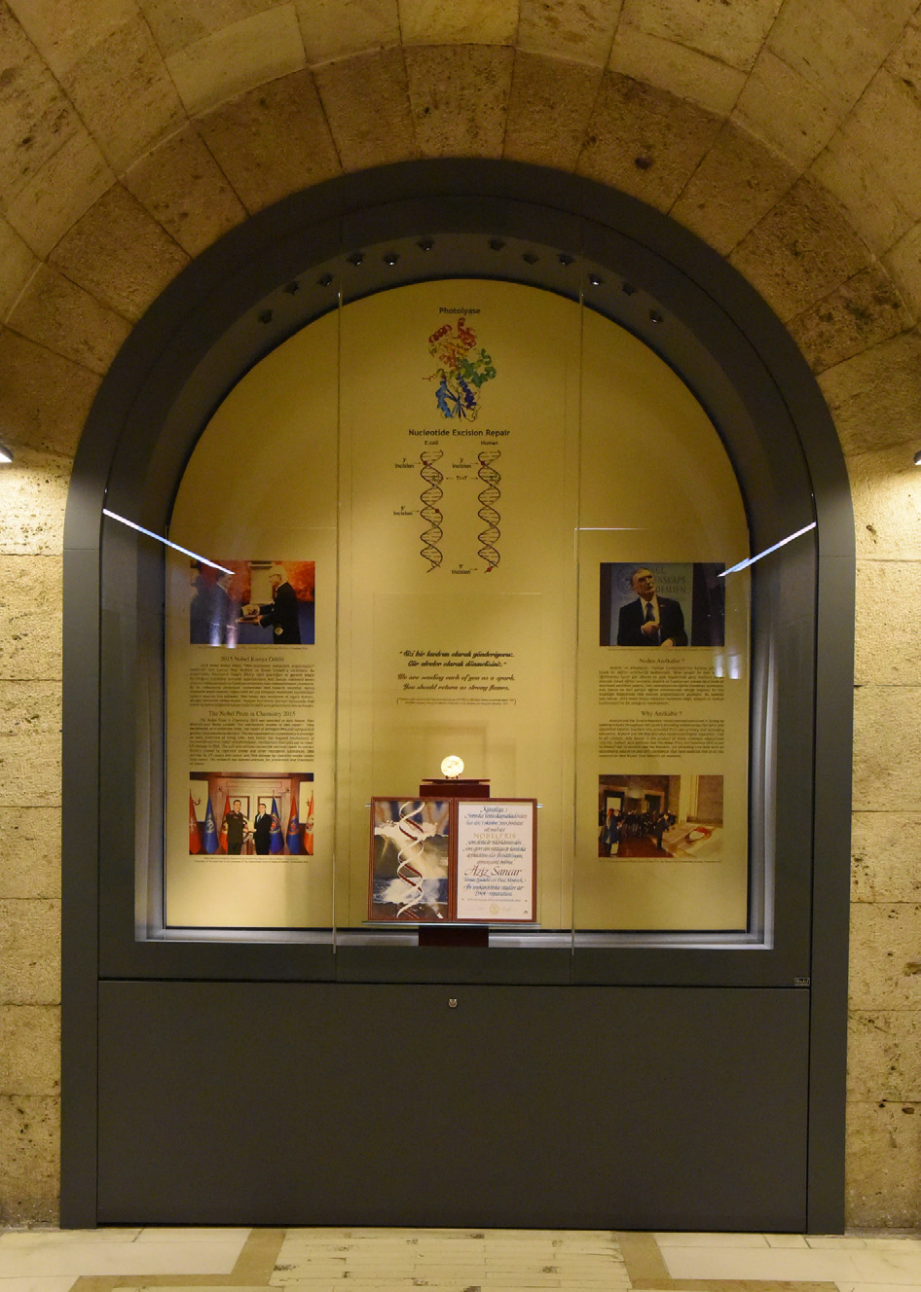
Nobel Prize in Chemistry 2015, in Anıtkabir.

Aziz Sancar's 2015 Nobel Prize in Chemistry medal and diploma is permanently displayed at Anıtkabir in Ankara, Turkey – the Nation's most important memorial and museum. Inside *The Atatürk and Independence War Museum*, there is a special corner for Aziz Sancar's Nobel medal, diploma, and the story of his scientific journey along with the 20th century history of science in Turkey to inspire the present and future generations (Fig. 4):

“Atatürk and the Turkish Republic revolutionized education in Turkey by opening schools throughout the country including remote areas like Savur and appointed very idealist teachers who provided first‐rate primary and secondary education. Atatürk and the Republic also modernized higher education, free to all citizens. Aziz Sancar is the product of those visionary educational reforms. Indeed, Aziz thinks that the Nobel Prize in Chemistry 2015 is not to himself but to Atatürk and the Republic, both providing him with an outstanding education and self‐confidence that have enabled him to do the research on DNA Repair that benefits all mankind.”

## Looking into the Future: Fact I

Medical genetics in Turkey should be viewed in the context of the rich migration history of the Middle East, the Balkans, and the Mediterranean basin. The region is home to approximately 10% of the world's population today and has been a central hub of human migration and population admixture. In a study published in the September 2016 issue of *Nature Genetics* (Scott et al. 2016), the genomic landscape of the region is revealed providing a comprehensive view of genetic variation for enhanced discovery of disease‐associated genes and comparisons with global ancestry populations. With the participation of research groups from Algeria, Egypt, France, Morocco, Iran, Iraq, Israel, Jordan, Lebanon, Libya, Pakistan, Qatar, Saudi Arabia, Syria, Tunisia, Turkey, United Arab Emirates, and United States, scientists first collected whole‐exome data and compared these with data from 1000 Genomes Project Populations. They detected tight clusters of European and Asian populations, and high levels of divergence among the Middle Eastern regions. While populations from northwest Africa, the Arabian Peninsula, Persia, and Pakistan were found to be the least admixed, Turkish Peninsula and Syrian Desert population data were consistent with higher levels of European admixture.

## Looking Into the Future: Fact II

Medical genetics studies since the early 1980s consistently indicated that consanguineous populations such as the ones from the Middle East facilitate Mendelian disease gene identification significantly (Özçelik et al. 2010). Highest levels of consanguinity in the world are observed on the southern and eastern rims of the Mediterranean basin, Middle East, Mesopotamia, the Gulf, and the Indian subcontinent to southeast Asia. The reasons for preferring consanguineous marriages lie in historical and contemporary cultural preferences such as maintenance of family structure and property, and financial advantages relating to dowry. Additional factors include better relations with in‐laws and the perception that intrafamilial marriages are more stable. Although there are regional differences even among single countries, the overall rate of first cousin marriages are around 25% in the region. When multiple layers of consanguinity originating from endogamy is considered, homozygosity rates increase beyond the predictions that are based on kinship alone, ultimately raising the levels of inbreeding coefficiency. The direct consequence of such a genetic structure is increase in the number of recessive diseases.

Importantly, beginning from the 1980s the genomic resources of the region significantly contributed to the global efforts centered on identification of disease‐associated genes. For example, genes associated with neurodevelopmental disorders (*ALS2*,* ATP8A2*,* FA2H*,* FGD4*,* HTRA2*,* SNAP29*,* VLDLR*,* VRK1*,* WDR81*) and rare diseases (*ALX1*,* BBS10*,* CHST14*,* DDR2*,* SLC34A2*,* TAC3*,* TACR3*) have been identified in Mediterranean and Middle Eastern families. This is coupled with excellent clinical medicine services and functional biobanks funded by regional and EU resources. For example, every major medical school in Turkey has a medical genetics department now. Several referral centers carry out exome sequencing for research and diagnostic purposes.

The exome‐wide view of the Mediterranean basin and the Middle East shows that estimated inbreeding coefficients are around 10‐ to 20‐fold higher than those in European, African, and East Asian 1000 Genomes Project populations. This translates to an impressive four‐ to sevenfold increase in the identification of potential disease‐causing variants for unsolved recessive conditions (Scott et al. 2016; Özçelik and Onat 2016).

## Reverse Phenotyping for Complex Diseases

**Figure 5:**
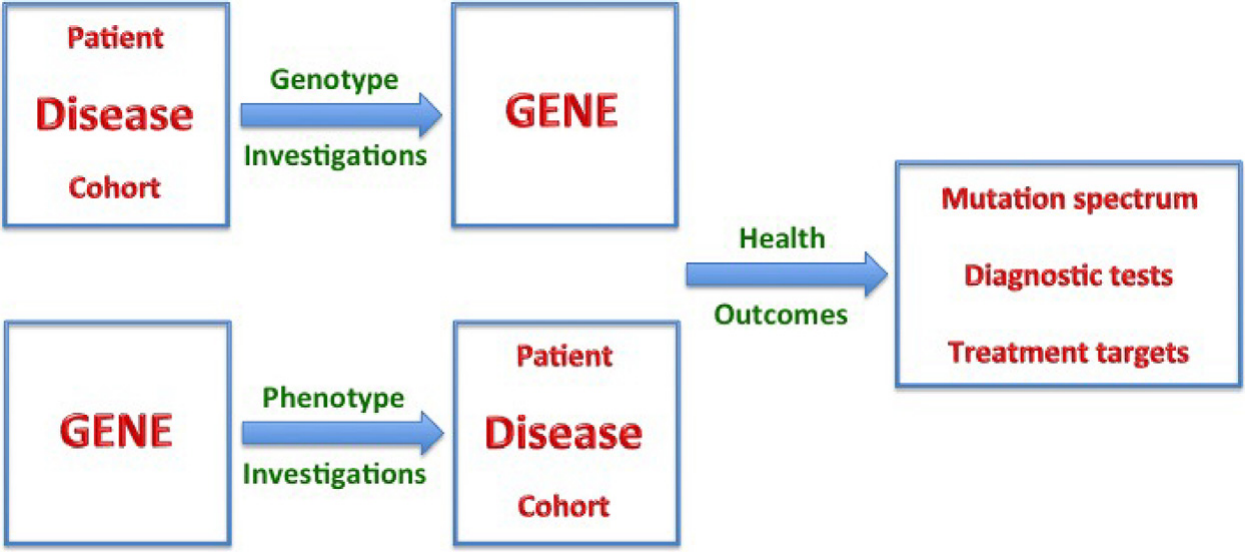
A scheme for “reverse phenotyping” of disease‐associated genes.

In the years following the adoption of next‐generation sequencing, an extreme degree of allelic, locus, and phenotypic heterogeneity has emerged for commonly observed diseases such as diabetes, extreme forms of obesity, hypertension, or neurodegeneration, making it notoriously difficult to resolve their molecular determinants at the genomic level. A scheme called “reverse phenotyping” offers an opportunity to facilitate the way forward in clinical delivery and informatics in the age of precision medicine (Özçelik and Onat 2016). Briefly, it consists of selection of candidate genes, prioritization of candidate variants from a cohort by filtering through prediction programs, phenotyping of individuals with candidate variants, establishment of causality by Mendelian segregation in large consanguineous families, and population screening (Fig. 5).

A successful application of reverse phenotyping recently occurred when the circadian rhythm gene cryptochrome 1 (*CRY1*) was found mutated in humans with delayed sleep phase disorder (DSPD) (Patke et al. 2017). Sleep disturbances is a common medical problem that affects nearly 10% of the population. Circadian disruption is known to predispose individuals to metabolic, cardiovascular, and psychiatric diseases, and lead to obesity and cancer.

## The Future is Now: The Turkish Genome Project

During the past decade, Turkey achieved universal health coverage and significant improvements in outcomes and equity, thus transforming the country's health system. This achievement is a good example that with commitment at the political level, middle‐income countries can simultaneously improve public health, financial risk protection, and user satisfaction (Atun 2015).

Armed with this experience, Turkey is now in the planning phase of an ambitious genome project that is centered on the identification of inherited gene mutations in rare as well as common diseases and cancer. The Turkish NIH, TÜSEB (Health Institutes of Turkey) initiated The Turkish Genome Project, which is planned in two phases: the first targeting 100,000 genomes in the next 3 years, and then reaching to 1,000,000 genomes before the 100th anniversary of the Republic in 2023. A major component of the project will be on complex phenotypes with high heritability index such as obesity, diabetes, cardiovascular, neuropsychiatric, endocrine, and rheumatologic diseases. A parallel‐executed component will focus on rare diseases, an area that Turkey has contributed significantly over the years. And yet another major component of the project is on cancer with particular emphasis on familial forms of these diseases.

## Conclusion: Genotheca Mediterranea

We witnessed during past three decades a spectacular success of scientists and families from Algeria, Cyprus, Egypt, France, Greece, Iran, Israel, Italy, Jordan, Lebanon, Morocco, Pakistan, Palestine, Spain, Tunisia, and Turkey working together and in collaboration with scientists from the EU and USA to uncover the genetic determinants of inherited diseases. In the words of renowned geneticist Giovanni Romeo, they built the foundations of a genomics‐based life sciences network they like to call “Genotheca Mediterranea,” akin to Bibliotheca Alexandrina. These success stories demonstrate the shared cultures and goals of scientists and the society on global problems of health and pave the way to future efforts to end human suffering.
